# The Causal Relationship Between Circulating Inflammatory Proteins and Tinnitus: A Mendelian Randomization Study Mediated by Blood Metabolites

**DOI:** 10.1002/brb3.70699

**Published:** 2025-09-02

**Authors:** Keyu Chen, Yuankun Cai

**Affiliations:** ^1^ Department of Neurosurgery Zhongnan Hospital Wuhan University Wuhan China

**Keywords:** blood metabolites, causal inference, inflammatory factors, Mendelian randomization, tinnitus

## Abstract

**Purpose:**

Tinnitus is a complex disease whose pathophysiological mechanisms are still not fully elucidated. Dysregulation of circulating inflammatory proteins and metabolites is thought to play a crucial role in tinnitus pathophysiology, but the causal relationships and specific biological pathways linking these factors to tinnitus persistence remain unestablished.

**Methods:**

We performed a two‐sample Mendelian randomization (MR) analysis using tinnitus genome‐wide association study (GWAS) data from FinnGen biobanking and GWAS data from metabolites and circulating inflammatory factors in GWAS directories. For the identified key metabolites, we further investigated their mediating role in the effects on tinnitus using mediator MR. We explored potential mechanisms through protein–protein interaction and functional enrichment analyses.

**Results:**

MR analysis showed that Chemokine (C‐C motif) Ligand 19 (CCL19) (OR = 1.071; 95% CI, 1.006–1.141; *p* = 0.032) was positively correlated with tinnitus. In addition, MR analysis identified 14 serum metabolites significantly associated with tinnitus. Among the metabolites, pantothenate (OR = 1.047; 95% CI, 1.012–1.082; *p* = 0.007) may play a critical mediating role in CCL19‐induced tinnitus. Protein–protein interaction and functional enrichment analysis showed that CCL19 may promote tinnitus through extensive interactions with genes related to pantothenic acid metabolism, such as Toll‐like receptor 4 (TLR4) and Scavenger Receptor Class B, Member 1 (SCARB1), by promoting oxidative stress and inflammation. And the downstream inflammation‐related pathways of pantothenate (such as Toll‐like receptor, NF‐κB, etc.).

**Conclusion:**

This first MR‐based evidence establishes CCL19‐pantothenate axis dysfunction as a causal driver of tinnitus, prioritizing these biomarkers for early detection. It also revealed the pivotal role of circulating inflammatory proteins and metabolic dysregulation in the pathogenesis of tinnitus. The findings provide new evidence‐based medical support for specific circulating inflammatory proteins and metabolites as biomarkers for early tinnitus prediction and risk assessment. They also offer valuable insights for subsequent research on tinnitus pathophysiological mechanisms and precision medicine applications.

## Graphical abstract

This Mendelian randomization study reveals that elevated CCL19 increases tinnitus risk via pantothenate‐mediated inflammation, implicating the CCL19‐pantothenate‐TLR4/NF‐κB axis as a causal pathway. Findings identify novel biomarkers for early tinnitus prediction and highlight metabolic‐inflammation interplay in pathogenesis.

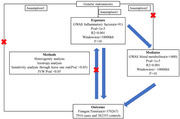

## 1 Introduction

Tinnitus is a subjective auditory sensation in the absence of auditory stimulation. Tinnitus can be divided into subjective tinnitus and objective tinnitus. Objective or somatic tinnitus originates from objective organic lesions such as head and face muscle movement or blood flow. However, subjective tinnitus perceives sound from no identifiable external or internal source. According to the latest global statistics, tinnitus affects more than 740 million adults worldwide, most of whom are older than 65 years of age, and more than 120 million people consider tinnitus to interfere with everyday life seriously (Jarach et al. 2022). Long‐term chronic tinnitus can cause severe pain, which can cause hyperacusis, insomnia, depression, anxiety, and many other problems (Yang et al. 2024). At present, tinnitus is considered to be mainly related to hearing impairment, but tinnitus can also occur in people without hearing impairment. The frequency of tinnitus is related to the degree and frequency of hearing loss, but the frequency of tinnitus and hearing loss do not entirely coincide (Liu et al. 2024). This suggests that, in addition to hearing loss, there may be other essential etiologies and mechanisms for the appearance of tinnitus that remain to be elucidated.

Recent animal experiments and population‐based case‐control studies have found that metabolite levels may be a pivotal contributor to tinnitus. Some studies have found significant changes in metabolite levels in the brain and serum of rats after auditory trauma through metabolomics analysis (He et al. 2021). A population‐based case‐control study found that the levels of circulating metabolites in patients with persistent tinnitus were significantly different from those in patients without a history of tinnitus, among which α‐keto‐β‐methylvaleric acid and levulinic acid were downregulated. At the same time, homocitrulline, three phosphatidylethanolamines, and two triglycerides were upregulated (Zeleznik et al. 2023). It has also been shown that endogenous dynorphin mediates enhanced glutamate excitability toxicity in cochlear Type I auditory dendrites, which may exacerbate chronic subjective nerve‐generated tinnitus during heightened stress (Sahley et al. 2013).

In addition to circulating metabolites, inflammation is also implicated in tinnitus pathophysiology. In animal models of tinnitus, levels of inflammatory cytokines (e.g., TNF‐α and IL‐1β) are increased throughout the auditory pathway, while microglia and astrocytes are also activated (X. H. Chen and Zheng 2017; Hu et al. 2014; Mennink et al. 2022; Tang et al. 2024). There are also cross‐sectional studies on tinnitus and healthy people with (near) normal hearing and no signs of depression or anxiety, which found that the concentration of IL‐10 and IFN‐γ cytokines in tinnitus participants was lower than that in the control group (Mennink et al. 2024). Recent studies have also found that the TLR4/NF‐κB/NLRP3 protein/caspase‐1/IL‐1β signaling pathway is essential in neuroinflammation in noise‐induced tinnitus mice (Luo et al. 2024).

The cochlea is particularly vulnerable to oxidative stress damage due to its unique anatomical structure and physiological properties, such as high metabolic activity and limited blood supply (Maniaci et al. 2024). Oxidative stress injury can trigger the inflammatory response in the cochlea, leading to the activation and recruitment of immune cells, which further leads to the upregulation of proinflammatory cytokines, such as TNF‐α, IL‐6, and IL‐1β (Kalinec et al. 2017). At the same time, activated immune cells can generate more reactive oxygen species (ROS), thus forming a complete feedback loop to aggravate cochlear damage further. Cochlear damage may eventually lead to hearing loss or tinnitus.

While some studies have investigated the potential involvement of inflammation or metabolic processes in the etiology of tinnitus, the precise role of the intricate interplay between these two mechanisms in tinnitus still needs to be better understood, particularly in light of the complex etiological factors involved.

MR analysis evaluates the causal relationship between exposure factors and outcomes using genetic variants as instrumental variables. Recently, it has become a powerful genetic epidemiological research method (Burgess et al. 2024). Compared with traditional observational research methods, MR analysis can provide more robust evidence for causal inference by using genetic variants randomly assigned at the interaction time, unaffected by confounding factors or reverse causality (Lovegrove et al. 2024). In recent years, MR analysis methods have been used to study the causal role of circulating biomarkers (such as trace elements, inflammatory factor proteins, or circulating metabolites) in various diseases, including tumors and hearing impairment (Ni et al. 2024; Zhou et al. 2023). However, no comprehensive MR study has systematically evaluated the causal effects of circulating inflammatory proteins and metabolites on tinnitus.

In this research, we employed a mediating MR design to investigate the potential mediating role of metabolites in the causal pathway from inflammatory proteins to tinnitus. Concurrently, we comprehensively evaluated the causal relationship between circulating inflammatory proteins, metabolites, and tinnitus risk. By combining genetic data, transcriptomics, proteomics, and metabolomics data, our objective is to elucidate the intricate molecular mechanisms of the inflammation–metabolism–tinnitus axis and identify novel targets and strategies for preventing, early detection, and treating tinnitus. Our results may offer new insights into the etiology and mechanisms of tinnitus and facilitate the development of precision medicine approaches for this recalcitrant disease.

## 2 Material and Methods

### 2.1 Study Design

A two‐sample Mendelian randomization (MR) analysis was performed using pooled GWAS data on blood metabolites, circulating inflammatory proteins, and tinnitus. To avoid sample overlap and ensure unbiased causal effects, MR analyses must satisfy three key assumptions: Genetic variables are strongly associated with exposure. The genetic variables used as instrumental variables for exposure must be independent of confounders associated with the selected exposure and outcome. Instrumental variables can only affect the outcome through exposure, but not through other ways, and are not directly related to the outcome. In addition, we performed a mediation MR analysis. Because mediation MR analysis can help us assess the role of mediating factors in the causal pathway between exposure factors and outcomes, by integrating the two‐sample MR and mediation MR approaches, we can obtain a more comprehensive understanding of the causal relationship between blood metabolites, inflammatory factors, and tinnitus, as well as their underlying mechanisms.

### 2.2 Data Sources

Three primary data sources were utilized in this study. First, tinnitus‐associated GWAS summary statistics can be found in the FinnGen biological samples library database (https://finngen.gitbook.io/documentation/data‐download). Data from 7914 tinnitus cases, 362353 controls, and 21 million SNPs were included, and the dataset ID was finn‐b‐H8_TINNITUS. Second, we, in the public databases from the GWAS directory (https://www.ebi.ac.uk/gwas/), won the 1400 metabolites GWAS results (ID: GCST90199621 to GCST90201020) (Y. Chen et al. 2023). At the same time, we integrated the from Cambridge University in the Department of Public Health and Primary Care protein research, circulating inflammatory protein GWAS data (https://www.phpc.cam.ac.uk/ceu/proteins). GWAS directory ID: GCST90274758 to GCST90274848 (Zhao et al. 2023). In addition, all study participants in the three databases were of European ancestry. Details of each data source are provided in Table S1.

### 2.3 Selection of Instrumental Variables

A genome‐wide significance threshold of *p* < 1 × 10^−5^ was set to ensure that the selected instrumental variables were vital. In addition, to ensure the independence of selected instrumental variables, we ruled out linkage disequilibrium (LD) with a physical distance threshold of 10,000 kb and an R2 threshold of 0.001. To further ensure the validity of instrumental variables, *F*‐statistics were used to assess their strength, which was calculated for all SNPS, and we eliminated SNPS with frontal *F*‐values less than 10. The *F*‐statistic was calculated as follows: *F* = beta 2/se 2, where beta represents the effect estimate of the exposure factor on the outcome variable, and so is the uncertainty measure of the effect estimate, reflecting the precision of the beta value. Details regarding eligible instrumental variables used for the MR analysis are provided in Table S2.

### 2.4 Statistical Analysis

Five MR analysis methods were used in this study, including inverse variance weighting (IVW), MR‐Egger regression, weighted median (WM), simple mode, and weighted mode. Considering that the IVW method provides the most accurate and unbiased estimation of the causal effect of exposure factors and outcomes, IVW was chosen as the primary method for causal effect determination.

We performed several sensitivity analyses to assess the heterogeneity and horizontal pleiotropy of the MR Results. First, Cochrane's *Q* test assessed heterogeneity, with *p *> 0.05 indicating no heterogeneity had been found. Second, horizontal pleiotropy was assessed by MR‐Egger intercept and Mendelian random polymorphism residuals and outliers (MR‐PRESSO) global tests, with *p *> 0.05 indicating the absence of significant horizontal pleiotropy. Leave‐one‐out tests were used to eliminate each SNP stepwise, calculate the combined effect of the remaining SNPS, and observe the influence and stability of individual SNPS on the results.

### 2.5 Mediation MR Analysis

Firstly, the total causal effect (*α*) of inflammatory factors on tinnitus was estimated by two‐sample MR. The dominant method was IVW, supplemented by MR‐Egger regression and WM for sensitivity analysis. Subsequently, two‐sample MR analyses were performed on inflammatory factors and metabolites and metabolites and tinnitus, respectively, to obtain causal effect estimates β1 and β2 while excluding genetic variants associated with inflammatory factors to maintain the validity of MR. Conditional analysis was performed by including inflammatory factors as covariates. Finally, the indirect effect β3 was obtained by calculating the product of β1 and β2, and the mediating effect proportion was determined by its ratio to α. Bootstrap methods were used to assess the statistical significance of mediating effects.

All MR analyses were performed using the R language (version 4.3.1) and associated R software packages, such as “TwoSampleMR” and “MRInstruments.”

## 3 Results

**FIGURE 1:**
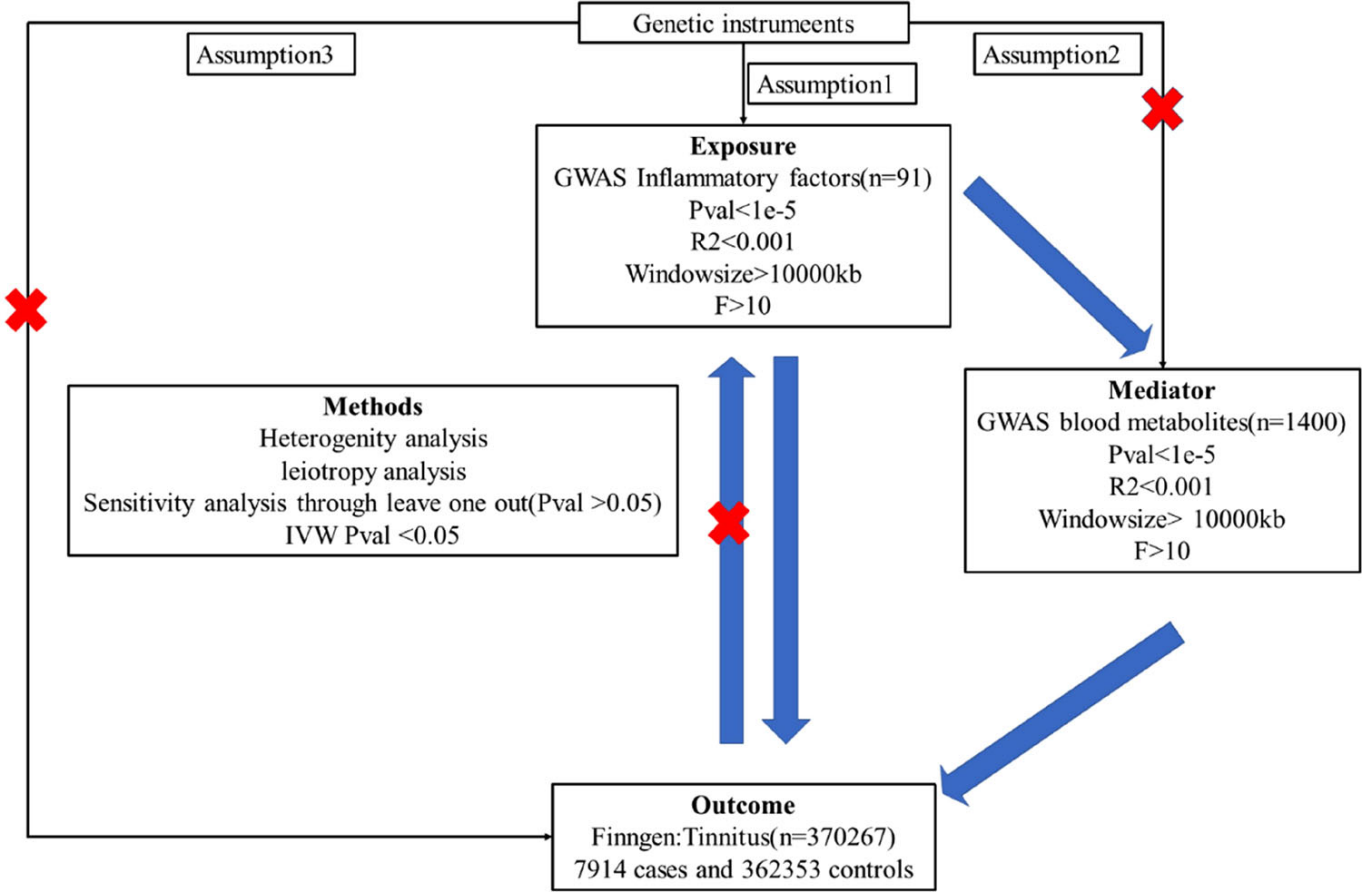
The study design and workflow of the present MR study.

All MR analysis procedures are shown in Figure 1.

### 3.1 Assessment of the Causal Relationship Between Circulating Inflammatory Factors and Tinnitus

**FIGURE 2:**
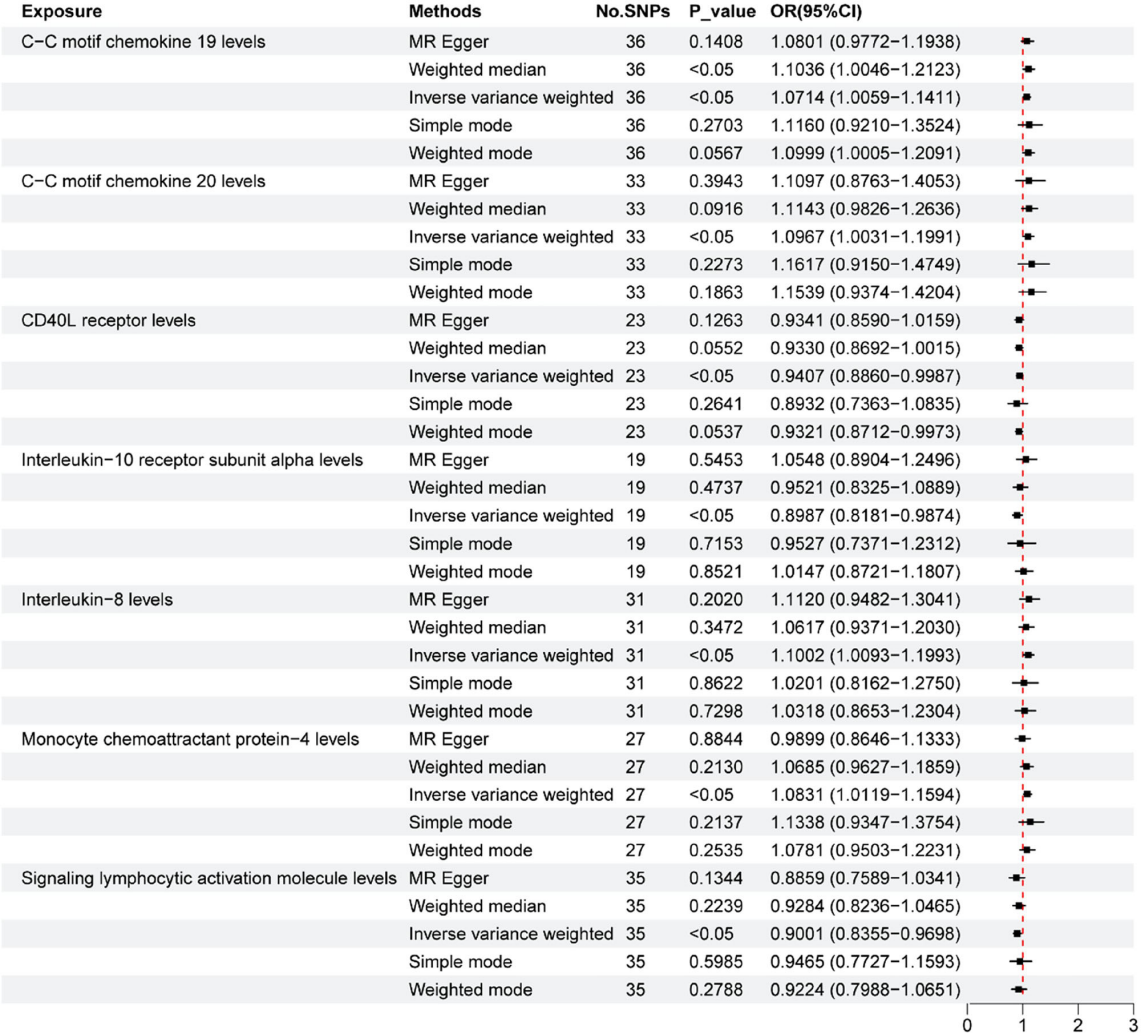
Forest plots of MR estimates of genetic causal associations between inflammatory cytokines and tinnitus. CCL19, C‐C motif chemokine 19; CCL20, C‐C motif chemokine 20; CD40L receptor, CD40 ligand receptor; CI, confidence interval; IL‐10Rα, interleukin‐10 receptor subunit alpha; IL‐8, interleukin‐8; IVW, inverse variance weighting; MCP4, monocyte chemotactic protein‐4; MR, Mendelian randomization; OR, odds ratio; SLAM, signaling lymphocytic activation molecule.

We used a two‐sample MR approach to assess the causal relationship between 91 circulating inflammatory factors and tinnitus. Preliminary results (Figure 2) showed that among all the inflammatory factors analyzed, C‐C motif chemokine ligand 19 (CCL19) (odds ratio [OR] = 1.071; 95% confidence interval [CI], 1.005–1.141; *p* = 0.032), C‐C motif chemokine 20 (CCL20) (OR = 1.096; 95% CI, 1.003–1.199; *p* = 0.032), C motif chemokine 20 (CCL20) (OR = 1.096; 95% CI, 1.003–1.199; *p* = 0.032). *p* = 0.042), interleukin‐8 (IL‐8) (OR = 1.1; 95% CI, 1.009–1.199; *p* = 0.042), and IL‐8 (OR = 1.1; 95% CI, 1.009–1.199; *p* = 0.042). *p* = 0.03) and monocyte chemotactic protein‐4 (MCP‐4) (OR = 1.083; 95% CI, 1.011–1.159; *p* = 0.021) were increased. CD40 ligand receptor (OR = 0.94; 95% CI, 0.886–0.998; *p* = 0.045), interleukin‐10 receptor subunit alpha (IL‐10Rα) (OR = 0.898; 95% CI, 0.818–0.987; *p* = 0.045), and IL‐10Rα (OR = 0.898; 95% CI, 0.818–0.987; *p* = 0.045). *p* = 0.026) and signaling lymphocytic activation molecule (SLAM) (OR = 0.9; 95% CI, 0.835–0.969; *p* = 0.005) were associated with an increased risk of tinnitus. In addition, eukaryotic translation initiation factor 4E‐binding protein 1 levels (4EBP‐1) (OR = 0.984; 95% CI, 0.97–0.998; *p* < 0.05) were significantly associated with tinnitus. *p* = 0.03).

CCL19 had statistically significant differences and was further identified as a risk factor for tinnitus according to the OR value (OR = 1.071; 95% CI, 1.005–1.141; *p* = 0.032). This result suggested that increased CCL19 levels may be causally related to an increased risk of tinnitus (*β* = 0.0689).

To confirm the robustness of our findings, we performed multiple sensitivity analyses (Figure S1). The results of the sensitivity analyses were consistent with the IVW method. The MR‐Egger regression and WM method also supported a positive causal relationship between CCL19 and tinnitus. In addition, the Cochran *Q* test and MR‐PRESSO analysis did not reveal any significant heterogeneity and pleiotropy bias (Table S3).

This study used reverse MR analysis to gain insight into the potential inverse association between tinnitus and circulating levels of inflammatory factors. Using IVW analysis, we determined that the incidence of tinnitus was associated with decreased 4EBP1 levels (OR = 0.984; 95% CI, 0.97–0.998; *p* = 0.03). To confirm the robustness of these associations, we performed analyses using four other methods and performed sensitivity analyses (Table S4). In addition, we used scatter plots and funnel plots to demonstrate the consistency and reliability of our findings visually (Figure S2), thereby further improving the validity of our results.

However, we did not find that tinnitus did not have a significant causal effect on CCL19 levels. This finding rules out the possibility that tinnitus causes changes in CCL19 levels and further supports a unidirectional causal effect of CCL19 on tinnitus.

### 3.2 Assessment of the Causal Relationship Between Metabolites and Tinnitus

**FIGURE 3:**
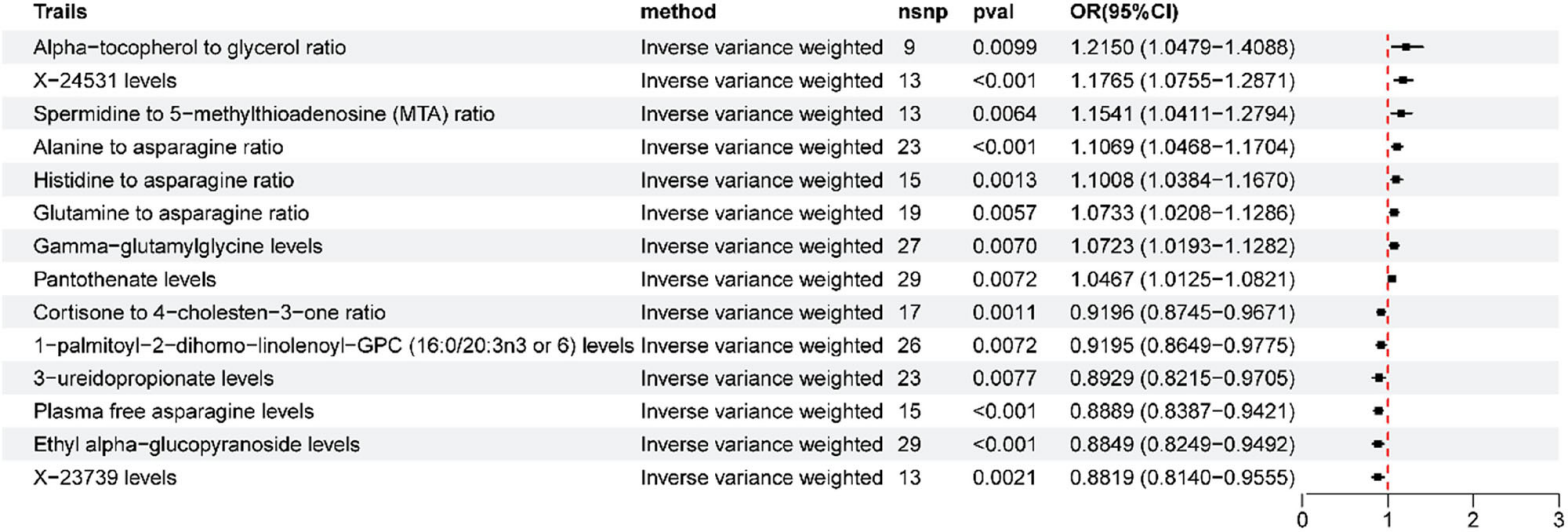
Forest plots of MR estimates of genetic causal associations between tinnitus and blood metabolites. 4EBP‐1, 4E‐binding protein 1; CI, confidence interval; IVW, inverse variance weighting; MR, Mendelian randomization; OR, odds ratio.

We used a two‐sample MR approach to assess the causal relationship between various metabolites and tinnitus (Figure 3). Some metabolites associated with a reduced risk of tinnitus were identified. These included X‐23739 (OR = 0.881908, 95% CI, 0.813993–0.955489), Ethyl alpha‐glucopyranoside (OR = 0.884901, 95% CI, 0.824927–0.949236), Plasma free asparagine (OR = 0.888908; 95% CI, 0.838691–0.942131), 3‐ureidopropionate (OR = 0.89289; 95% CI, 0.821509–0.970474), 1‐palmitoyl‐2‐dihomo‐linolenoyl‐GPC (16:0/20:3n3 or 6) (OR = 0.919509; 95% CI, 0.864934–0.977527), and the cortisone to 4‐cholesterin‐3‐one ratio (OR = 0.919615; 95% CI, 0.874473–0.967088), Meanwhile, some metabolites, such as Pantothenate (OR = 1.046718; 95% CI, 1.012454–1.082142), Gamma‐glutamyl glycine (OR = 1.07235; 95% CI, 1.019252–1.128214), Glutamine to asparagine ratio (OR = 1.073349; 95% CI, 1.038419–1.166961)

Histidine to asparagine ratio (OR = 1.100815; 95% CI, 1.038419–1.166961), alanine to asparagine ratio (OR = 1.100815; 95% CI, 1.038419–1.166961), histidine to asparagine ratio (OR = 1.106914; 95% CI, 1.046843–1.170432), spermidine to 5‐methylthioadenosine (MTA) ratio (OR = 1.154144; 95% CI, 1.041143–1.279411), X‐24531 (OR = 1.176534; 95% CI, 1.075451–1.287117), and alpha‐tocopherol to glycerol ratio (OR = 1.215038; 95% CI, 1.047929–1.408794). This variety may be associated with an increased risk of tinnitus. The results of the heterogeneity and pleiotropy analyses for each metabolite and tinnitus are presented in Table S5.

### 3.3 Assessment of Causal Relationships Between Circulating Inflammatory Factors and Metabolites

To further analyze the possible mediating effect of metabolites in the relationship between inflammatory factors and tinnitus, the present study examined the causal link between CCL19, an inflammatory factor associated with increased risk of tinnitus in previous studies, and specific metabolites. Using two‐sample MR analysis, we considered CCL19 as a potential exposure and metabolites as study endpoints to explore the causal association between CCL19 and metabolites.

**FIGURE 4:**
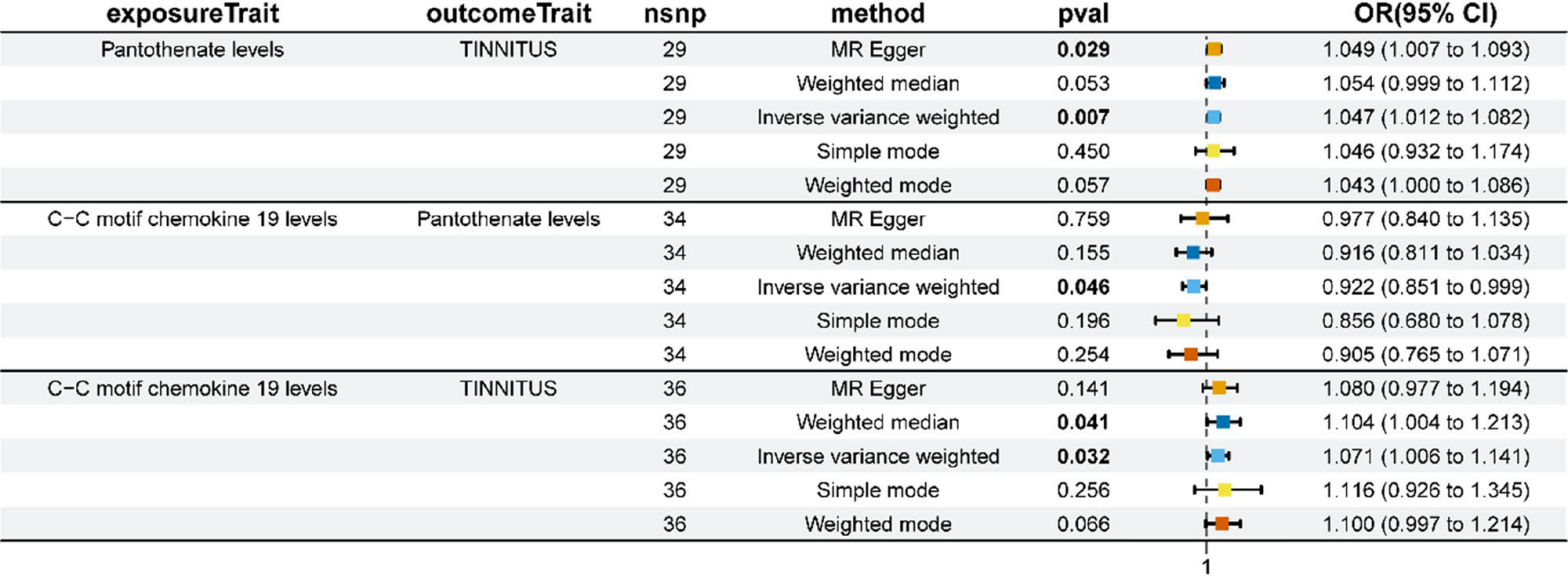
Forest plot of MR analysis of the causal relationship between CCL19 and pantothenic acid on tinnitus and the causal relationship between CCL19 and pantothenic acid.

The results of the IVW analysis in Figure 4 revealed a significant positive causal association between CCL19 and pantothenate levels (OR = 1.071; 95% CI, 1.006–1.141; *p* = 0.032). This suggested that the increase in CCL19 level might induce the increase of pantothenate level (estimated coefficient *β*1 = −0.1169). Taken together with the positive association between pantothenate levels and tinnitus risk found in previous studies (OR = 1.047; 95% CI, 1.012–1.082, *p* = 0.007), this new finding points to a possible mediating role of pantothenate in the effect of CCL19 on tinnitus. In other words, CCL19 may increase the risk of tinnitus by increasing the level of pantothenate. Other aspects of the analysis, including heterogeneity and pleiotropy, as well as the specific content of sensitivity analyses, are presented in detail in Table S6.

Nevertheless, no notable causal correlation was identified between CCL19 and the remaining metabolites (*p* > 0.05). Furthermore, no significant causal relationship was identified between the other circulating inflammatory factors and metabolites. This indicates that metabolites may serve an indispensable mediating function solely within the CCL19 pathway and that their involvement in the pathogenesis of other inflammatory factors in tinnitus is constrained.

### 3.4 Functional Inference of Pantothenate‐Related Gene Metabolic Network by CCL19

**FIGURE 5:**
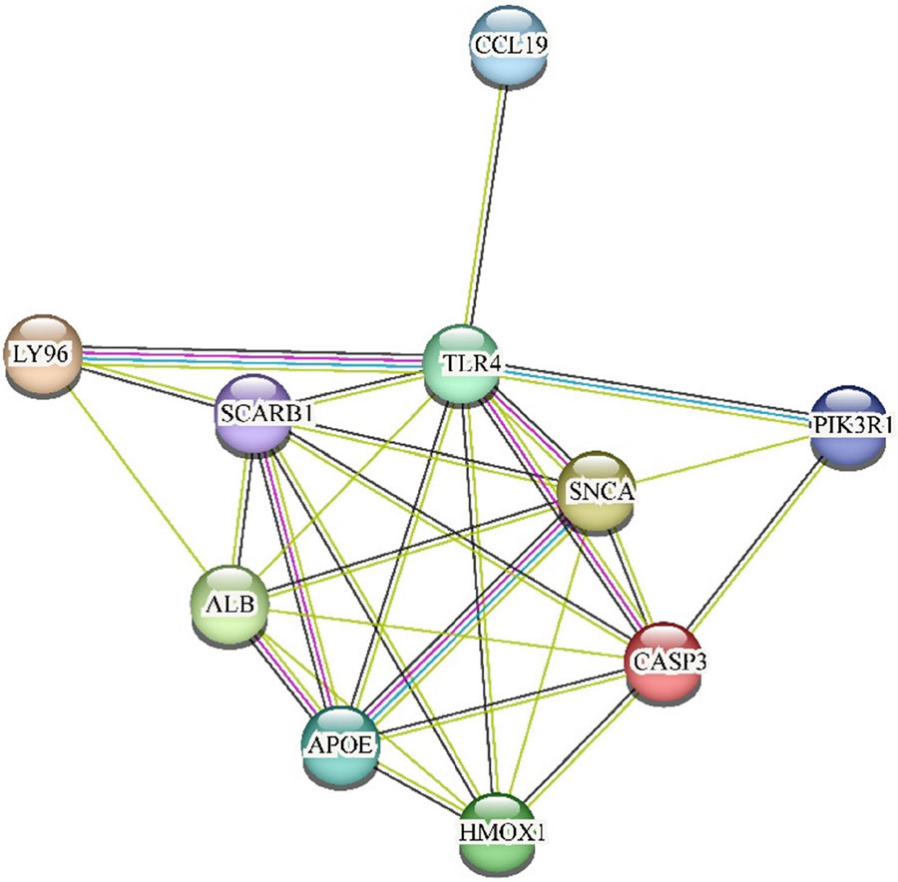
PPI network of CCL19 and pantothenic acid‐related genes.

The results of previous MR analyses suggest that CCL19 may promote the development and progression of tinnitus by increasing pantothenate levels. In order to dig deeper into the potential molecular mechanism of action, this study used the GeneCards database (https://www.genecards.org/) to retrieve the pantothenate‐related genes and build the protein between these genes and CCL19–protein interaction (PPI) networks (see Figure 5). The results showed that pantothenate‐related genes had a wide range of intracellular interactions with CCL19, suggesting that CCL19 may affect the level of pantothenate by regulating the expression or function of these genes. Detection of lipopolysaccharide plasma lipoprotein particle clearance positively regulates nerve cell death, regulation of nerve cell death regulates tumor necrosis factor production, regulation of monooxygenase activity, regulation of oxidoreductase activity, response to lipopolysaccharide high‐density lipoprotein particle clearance isassociated with inflammatory response, wound healing.

**FIGURE 6:**
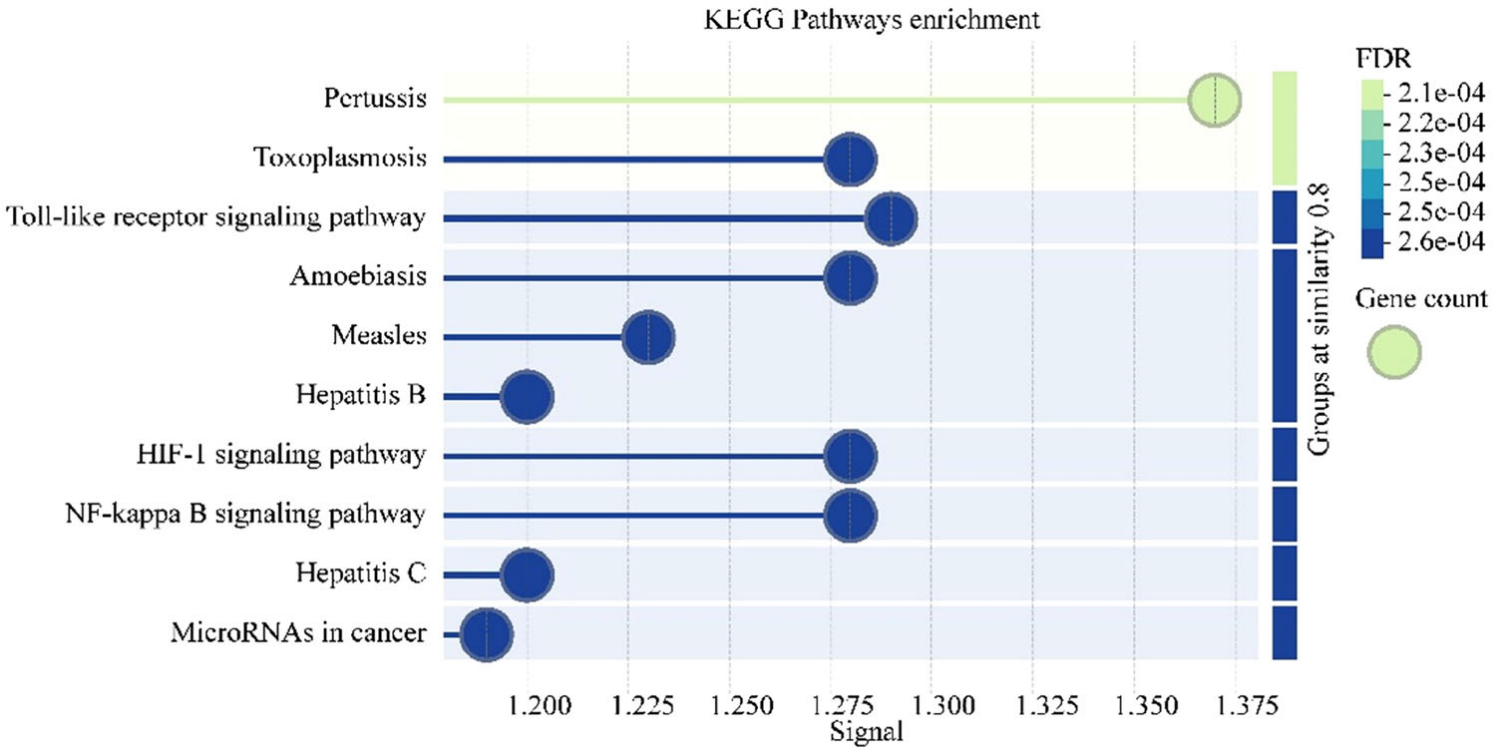
GO functional enrichment bubble map of CCL19‐related genes.

To explore the functional characteristics of pantothenate regulated by CCL19, Gene Ontology (GO) and Kyoto Encyclopedia of Genes and Genomes (KEGG) pathway enrichment analysis were performed on the genes in the protein–protein interaction network. The results of GO analysis, shown in Figure 6, revealed that these genes were significantly enriched in multiple biological processes, including inflammatory responses (e.g., response to lipopolysaccharide and tumor necrosis factor production), metabolic dysregulation (e.g., clearance of plasma lipoprotein particles and high‐density lipoprotein particles), neurodegenerative changes (e.g., positive regulation of nerve cell death, P. “They may be associated with neurological disorders and development”), and oxidative stress responses (e.g., modulation of monooxygenase and oxidoreductase activity).

This suggests that CCL19 may promote the occurrence and development of tinnitus by promoting oxidative stress and inflammatory responses involving pantothenate‐related genes and influencing the response process to inflammatory factors, thereby remodeling the related auditory nerve circuit.

**FIGURE 7:**
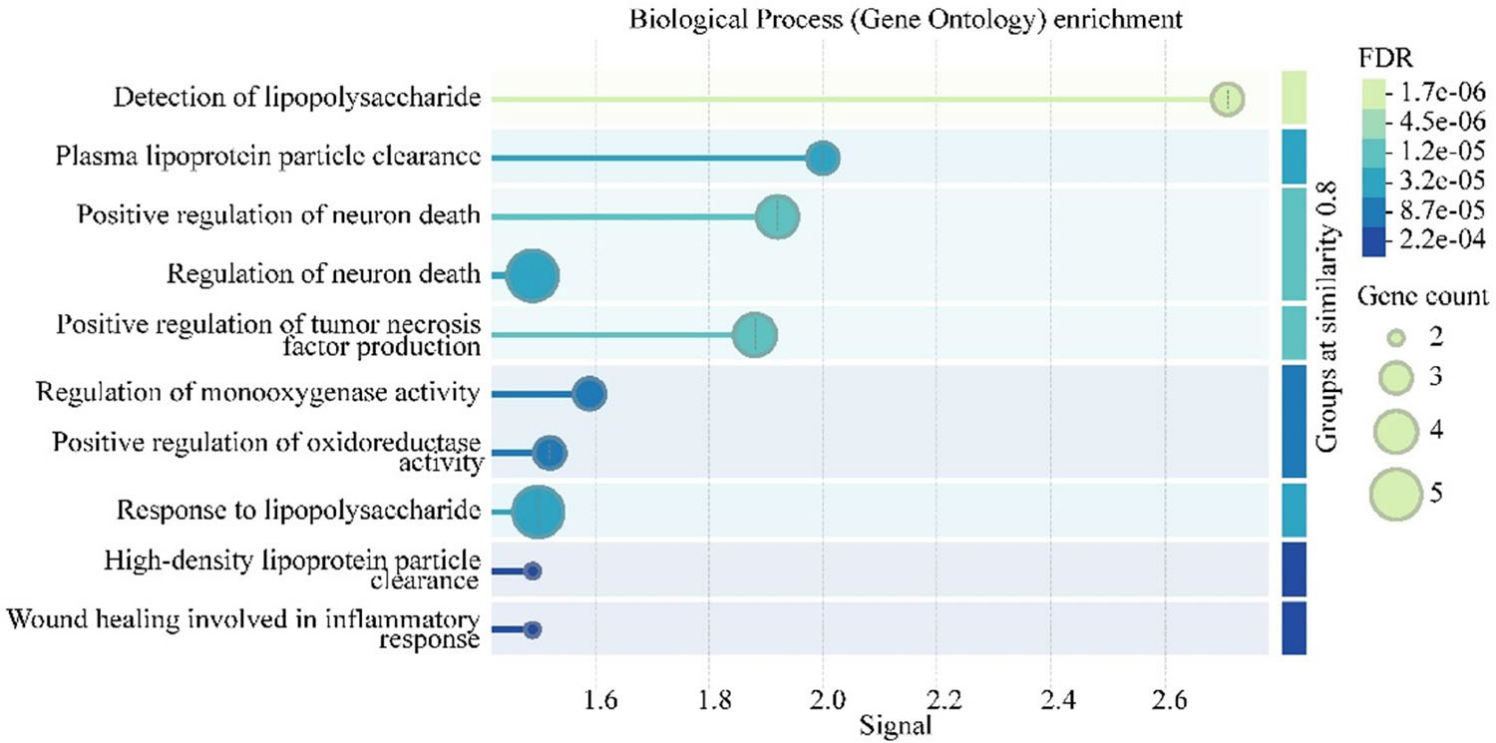
KEGG pathway enrichment map of pantothenic acid‐related genes.

KEGG pathway enrichment analysis (Figure 7) showed that pantothenate‐related genes were mainly enriched in disease‐related pathways. Such as pertussis, toxoplasmosis, amoebiasis, measles, and other infectious diseases. Immune and inflammation‐related pathways: Toll‐like receptor signaling pathway, NF‐kappa B signaling pathway, HIF‐1 signaling pathway, and other pathways. This suggests that CCL19 may promote tinnitus by activating these signaling pathways, thereby increasing pantothenate levels.

## 4 Discussion

In this study, we systematically reviewed potential causal associations between circulating inflammatory factors, serum metabolites, and tinnitus using MR. A total of 91 circulating inflammatory factors and 1400 serum metabolites were screened, and 7 inflammatory factors and 14 serum metabolites were found to have possible causal associations with tinnitus. CCL19 was positively correlated with tinnitus risk (OR = 1.071, 95% CI, 1.005–1.141, *p* = 0.032), suggesting that CCL19 may be a risk factor for tinnitus. We then used the same approach to explore the causal relationship between serum metabolites and tinnitus. We identified several metabolites that might reduce the risk of tinnitus, such as X‐23739, Ethyl alpha‐glucopyranoside, etc.

In addition, we identified metabolites that may be associated with an increased risk of tinnitus. These included pantothenate, gamma‐glutamyl glycine, glutamine to asparagine ratio, histidine to asparagine ratio, alanine to asparagine ratio, Histidine to asparagine ratio, spermidine to 5‐methylthioadenosine (MTA) ratio, X‐24531, and alpha‐tocopherol to glycerol ratio. To further explore the mechanism of CCL19 in promoting tinnitus, the causal relationship between CCL19 and these metabolites was analyzed using a mediated MR approach. The results showed that CCL19 significantly increased the level of pantothenate (OR = 1.071; 95% CI, 1.006–1.141; *p* = 0.032), which was consistent with the increased risk of tinnitus, suggesting that pantothenate may play an essential role in CCL19‐induced tinnitus. Further, by protein–protein interaction network and functional enrichment analysis, we found that CCL19 might promote tinnitus by exacerbating oxidative stress, inflammatory response, and inflammatory signaling pathways involving pantothenate‐related genes.

CCL19 is the gene encoding proinflammatory chemokine ligands important in recruiting normal lymphocytes or activated T cells from the peripheral blood to local areas of injury during trauma and acute noise stress (Zhang et al. 2018). It has been found that the CCL19 gene is significantly different after noise exposure in GRAIL KO mice compared with WT mice, and it is suggested that downregulation of CCL19 may disrupt signaling to immune cells and reduce the induction of inflammatory cytokines. Thus, inflammatory cells are recruited to the noise‐damaged cochlea to counteract the noise‐induced hearing loss in GRAIL KO mice (Chuang et al. 2023). In addition, elevated circulating levels of CCL19 are also present in various infections (Tveita et al. 2022).

The role of CCL19 in tinnitus has yet to be reported in the literature. However, the results of the present study provided strong evidence for its role in promoting tinnitus. This novel finding not only reveals the importance of CCL19 in the pathological mechanism of tinnitus but also provides a theoretical basis for using CCL19 as a potential specific target for treating and preventing tinnitus.

With the development of liquid chromatography and mass spectrometry technology, metabolomics research is becoming increasingly widely used in the field of tinnitus. Some studies used complementary liquid chromatography‐tandem mass spectrometry to analyze plasma metabolites of participants with persistent tinnitus and control participants, and the results showed that α‐keto‐β‐methylvalonate and propionate were negatively correlated with persistent tinnitus. In contrast, metabolites such as triglycerides were positively correlated with tinnitus (Zeleznik et al. 2023). Some studies have also used gas chromatography/mass spectrometry and immunoassay to quantify circulating steroids in patients with tinnitus and found that steroid levels were negatively correlated with tinnitus. This correlation was considered to be related to hypothalamic–adrenal axis dysfunction (Chrbolka et al. 2017).

At the animal level, studies have found enhanced purine metabolism, oxidative phosphorylation, and phosphorylation in prominent glutamatergic pathways in the anterior cingulate cortex tissue of tinnitus mice and also found a significant increase in functional connectivity between the anterior cingulate cortex and the primary auditory cortex in tinnitus patients, which is positively correlated with serum glutamate levels (Fan et al. 2024). Some studies have found significant metabolic changes in both brain and serum samples of mice with auditory traumatic tinnitus, affecting many metabolic pathways (He et al. 2021). In the peripheral auditory system, some studies have found that there are also significant differences in metabolite levels in the cochlear nucleus of rats at different maturation stages (Tan et al. 2022).

The results of the previous studies have shown that the metabolite profiles in tissue and serum samples are closely related to the occurrence and progression of tinnitus. However, distinguishing between metabolite changes as a cause or consequence of tinnitus is challenging because most previous studies have used case‐control designs. In addition, factors such as small sample sizes and differences in metabolomics platforms limit the reproducibility and generalizations of the results of previous studies.

In contrast to existing studies, this study used a two‐sample MR approach to systematically evaluate the causal relationship between 1400 serum metabolites and tinnitus. The results showed that changes in the levels of multiple metabolites were significantly associated with tinnitus risk. Regarding amino acid metabolism, we found that free asparagine levels and 3‐uretypropionic acid levels were significantly associated with tinnitus risk. Notably, asparagine is a precursor of the neurotransmitter glutamate; elevated glutamate levels in the central nervous system are associated with tinnitus, and glutamate excitotoxicity is a potential risk factor for tinnitus (Zemaitis et al. 2021). Studies have found that the glutamate level in the auditory cortex of rats exposed to noise is significantly increased, and the expression of glutamate transporter 1 is damaged after noise exposure (Cao et al. 2024). Some studies have also found significant differences in glutamate and γ‐aminobutyric acid metabolism in different brain regions of the auditory pathway in animal models of tinnitus by imaging techniques. This regional inhibition of glutamate metabolism reflected changes in the balance between excitability and inhibition in chronic tinnitus. This excitatory and inhibitory auditory pathway imbalance is also a therapeutic target for tinnitus (Brozoski et al. 2012; Isler et al. 2022; Ma et al. 2020).

There are relatively few studies on 3‐uretypropionic acid. These findings suggest that amino acid metabolism disorders may promote the occurrence and development of tinnitus through multiple processes, such as regulating the level and transduction of neurotransmitter signals.

Regarding lipid metabolism, we present a lipid metabolite, 1‐palmitoyl‐2‐dihomo‐linolenoyl‐GPC (16:0/20:3n3 or 6), significantly associated with tinnitus risk. 1‐palmitoyl‐2‐dihomo‐linolenoyl‐GPC, a phosphatidylcholine (PC) with a specific fatty acid chain composition, is a cell membrane component that can promote signal transduction, act as a free radical scavenger, and protect membrane lipids from oxidation (Braverman and Moser 2012). The role of 1‐palmitoyl‐2‐dihomo‐linolenoyl‐GPC in tinnitus remains unclear. However, studies in rats suggest that PC may help protect cochlear mitochondrial function (Seidman et al. 2002). Further investigation of the protective effects of PC derivatives in tinnitus may yield promising findings.

These findings suggest that abnormal lipid metabolism may play a central role in the pathogenesis of tinnitus. Its mechanism may influence the structure and function of cell membranes and the regulation of mitochondria‐related signal transduction pathways.

Regarding nucleotide metabolism, we found that the spermidine to 5‐methylthioadenosine (MTA) ratio was significantly associated with tinnitus risk. These metabolites are products of polyamine synthesis and inhibit the transmethylation reaction (Frau et al. 2013).

We also found that pantothenic acid levels were significantly associated with tinnitus risk. Pantothenic acid is a water‐soluble B vitamin. As part of coenzyme A (CoA) and acyl carrier proteins (ACP), pantothenic acid is essential for a large number of metabolic reactions (Freese et al. 2023). Previous studies have found a protective effect of pantothenic acid against drug‐induced ototoxicity in guinea pigs (Martínez Martínez et al. 1997). Pantothenic acid is also an essential trace element for the synthesis of acetyl‐CoA. Previous studies have found that pantothenic acid is significantly reduced in multiple brain regions in patients with a variety of neurodegenerative diseases, such as Parkinson's disease and Huntington's disease (Scholefield et al. 2021). In the brain, pantothenic acid is mainly located in myelin‐containing structures, and pantothenic acid is also involved in synthesizing fatty acids required for myelination. Pantothenic acid may have an antioxidant effect during inflammation. Elevated C‐reactive protein plays a vital role in the early stages of inflammation. A population‐based observational study found an inverse association between dietary pantothenic acid intake and subsequent C‐reactive protein concentration (Jung et al. 2017). Pantothenic acid and the inflammatory response are also inseparable. The link between pantothenic acid and inflammatory response involves Ach signaling, which is also involved in vagal cholinergic and splenic sympathetic anti‐inflammatory pathways (Bonaz et al. 2016; Cox et al. 2020). Mitochondrial AcCoA is required for Ach synthesis (Jope and Jenden 1980).

In the present study, we also observed a significant association between altered proportions of multiple metabolites and tinnitus risk. In particular, increases in the cortisone to 4‐cholesten‐3‐one ratio and alpha‐tocopherol to glycerol ratio were significantly associated with reduced tinnitus risk. These data suggest that the balance of ratios between metabolites may play an essential role in tinnitus pathophysiology and that altered metabolic ratios may indicate dysfunction of specific metabolic pathways.

Further analysis showed that CCL19 may indirectly promote the development of tinnitus by increasing the level of pantothenic acid. This not only suggests a facilitative role of CCL19 in the pathogenesis of tinnitus but also points to pantothenic acid as a possible key downstream mediator of the pro‐tinnitus effect of CCL19.

Subsequent bioinformatics analysis revealed an extensive protein–protein interaction and co‐expression network between CCL19 and pantothenic acid metabolism genes, such as SCARB1 and LY96. These genes were significantly enriched in biological processes involved in the occurrence and development of tinnitus, such as oxidative stress and inflammatory response. Among them, the enzymes encoded by genes such as TLR4 are involved in the metabolism of endogenous and exogenous compounds, and their increased activity may lead to DNA damage, cell apoptosis, and other pro‐tumor effects. As a proinflammatory factor, CCL19 may enhance oxidative stress and inflammatory responses by activating these genes, promoting tinnitus.

This inference is in line with the results of previous studies, but the specific molecular mechanism of CCL19 action still needs to be experimentally verified. CCL19 may directly regulate genes involved in pantothenic acid metabolism and promote tinnitus by activating downstream signaling pathways. Previous studies have shown that pantothenic acid and its derivatives can promote the proliferation and invasion of tumor cells through MAPK and NF‐κB pathways, while CCL19 can inhibit these pathways. Thus, CCL19 may increase pantothenic acid levels and activate its downstream pro‐tinnitus signaling pathway. This hypothesis needs to be further tested by subsequent cell and animal experiments.

Although this study initially revealed the causal relationship between inflammatory factors and metabolites and tinnitus, some limitations and potential biases remain that need to be further explored. First, the data were obtained from persons of European ancestry, which helps to minimize bias due to population differences. However, it is essential to assess whether the results are generalizable to other ethnic groups and populations. Second, although this study used multiple independent GWAS datasets and performed exhaustive sensitivity analyses, we still could not completely rule out the possible horizontal pleiotropy between genetic instrumental variables and tinnitus and the potential problem of weak instrument bias. In addition, the omics data used in this study are from a European population, and the experimental group's sample size is still relatively small compared to the control group. Limited by our sample size, we could only identify significant associations of a few metabolites with tinnitus risk. Finally, the tinnitus data sources used in this study did not classify tinnitus, so only an overall estimate can be made.

Future studies with cross‐validation using multiple metabolomics platforms in larger sample sizes and different populations are needed to obtain more robust and comprehensive results. Integrating metabolomics with other omics data, such as proteomics and transcriptomics, and constructing multi‐omics integration models will also help better understand metabolite changes' role in tinnitus pathogenesis. Finally, it is worth noting that although we initially explored the molecular mechanism of the interaction between CCL19 and pantothenic acid by bioinformatics analysis, these results are mainly based on published literature and database information and still need experimental validation in cells and animal models. Future studies can use techniques such as gene editing and RNA interference to manipulate the expression of CCL19 and pantothenic acid‐related genes in mouse models of tinnitus, observe their effects on the occurrence and progression of tinnitus, and explore the specific molecular mechanisms of their interaction. Integrating multi‐omics data and constructing the regulatory network of CCL19 and pantothenic acid in tinnitus will help to understand their functions and mechanisms of action more comprehensively.

## 5 Conclusion

Through two‐sample MR and bioinformatics analysis, this study revealed a causal relationship between inflammatory factors, serum metabolites, and tinnitus; highlighted the importance of inflammation and metabolic disorders in the pathological mechanism of tinnitus; and highlighted the critical role of CCL19 and pantothenic acid. The results provide biomarkers for early prediction and risk assessment of tinnitus, pointing out the direction for mechanism research and precision medicine application. Based on this, future studies can deeply analyze the molecular mechanism of related metabolites, construct a molecular network model of tinnitus, and develop CCL19 and pantothenic acid‐based intervention strategies to improve the prevention and treatment of tinnitus.

## Author Contributions

**Keyu Chen**: conceptualization, writing–review and editing, writing–original draft, methodology, software, data curation, formal analysis, visualization. **Yuankun Cai**: writing–review and editing, conceptualization, methodology.

## Ethics Statement

The data in this study were derived from published studies. This study met ethical standards and was approved by the Medical Ethics Committee of Wuhan University.

## Conflicts of Interest

The authors declare no conflicts of interest.

## Peer Review

The peer review history for this article is available at https://publons.com/publon/10.1002/brb3.70699.

## Supporting information




**Supporting Fig.1a**: Scatter plots of the causal association between inflammatory cytokines and tinnitus using an MR study.
**Supporting Fig.1b**: Funnel plots of the causal association between inflammatory cytokines and tinnitus using an MR study.
**Supporting Fig.1c**: The leave‐one‐out analysis of causal impacts of inflammatory cytokines on tinnitus.
**Supporting Fig.2a**: Scatter plots of the causal association between tinnitus and inflammatory cytokines using an MR study.
**Supporting Fig.2b**: Funnel plots of the causal association between tinnitus and inflammatory cytokines using an MR study.
**Supporting Fig.2c**: The leave‐one‐out analysis of causal impacts of tinnitus on inflammatory cytokines.

**Table S1**. Baseline characteristics of the study population
**Table S2**. Information of SNPs for each inflammatory protein as the exposure
**Table S3**. Summary statistics of the causal estimates of inflammatory proteins on the risk of tinnitus.
**Table S4**. Summary statistics of the causal relationship between tinnitus and levels of circulating inflammatory factors
**Table S5**. Summary statistics of the causal estimates of serum metabolites on the risk of tinnitus.
**Table S6**. Total effect statistical analysis of Mendelian randomization analysis of inflammatory factors and diseases.

## Data Availability

The data that support the findings of this study are available from the corresponding author upon reasonable request. The publicly available datasets used in the present study are available for consultation in the article and its Supporting Information.
