# Supplementary material for: The Causal Relationship Between Circulating Inflammatory Proteins and Tinnitus: A Mendelian Randomization Study Mediated by Blood Metabolites
Source: Brain Behav. 2025 Sep 2;15(9):e70699. doi: 10.1002/brb3.70699 (PMC12405592; doi:10.1002/brb3.70699)

**Supplementary Figure 1**

**Supplementary Figure S1a:** Scatter plots of the causal association inflammatory cytokines and tinnitus using MR study. The two-sample MR analyses were conducted using the MR-Egger, Inverse variance weighted, weighted median, simple mode and weighted mode. The estimated MR effect per method is depicted by the slope of each line.


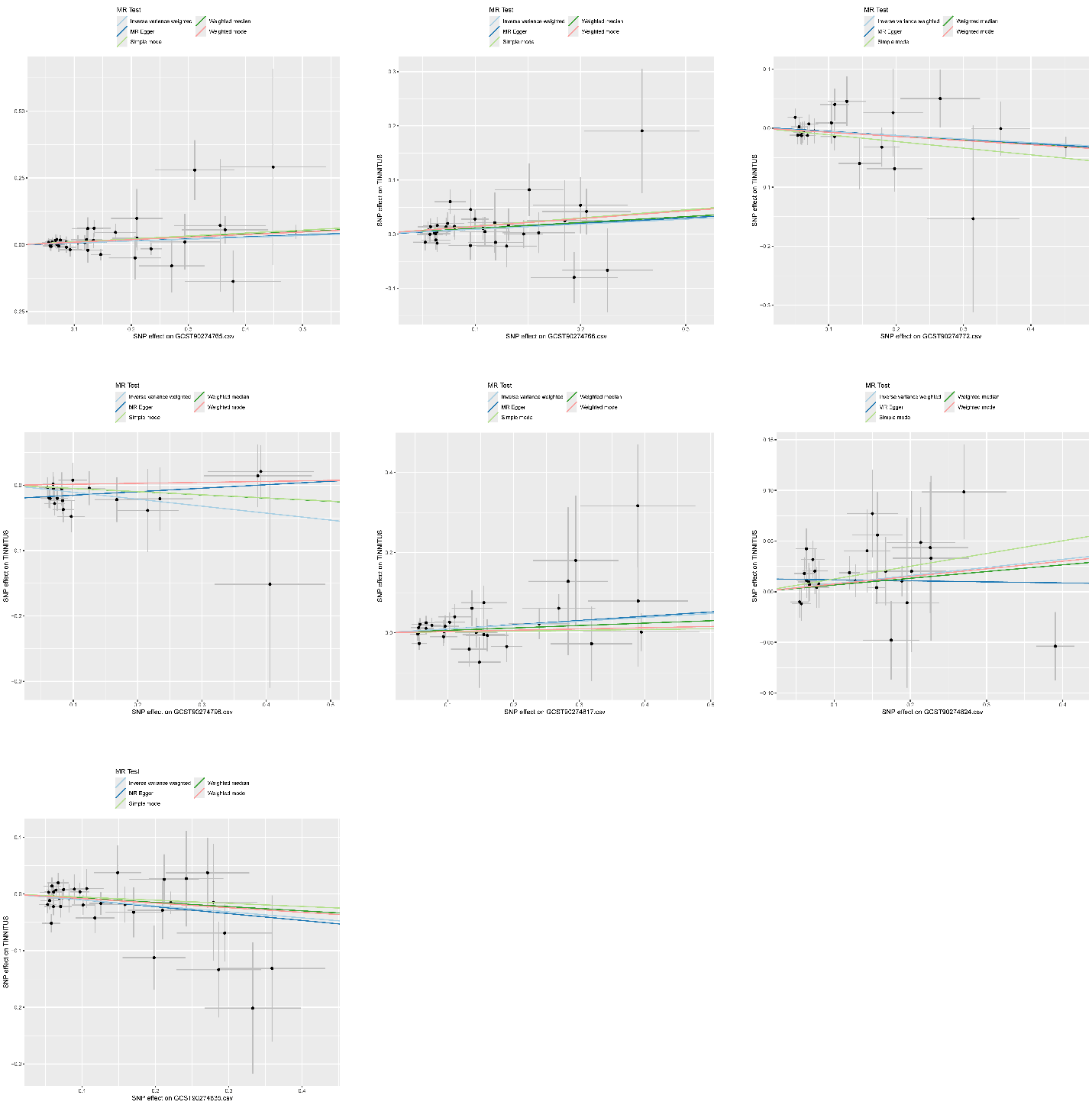


**Supplementary Figure S1b:** Funnel plots of the causal association between inflammatory cytokines and tinnitus using MR study.


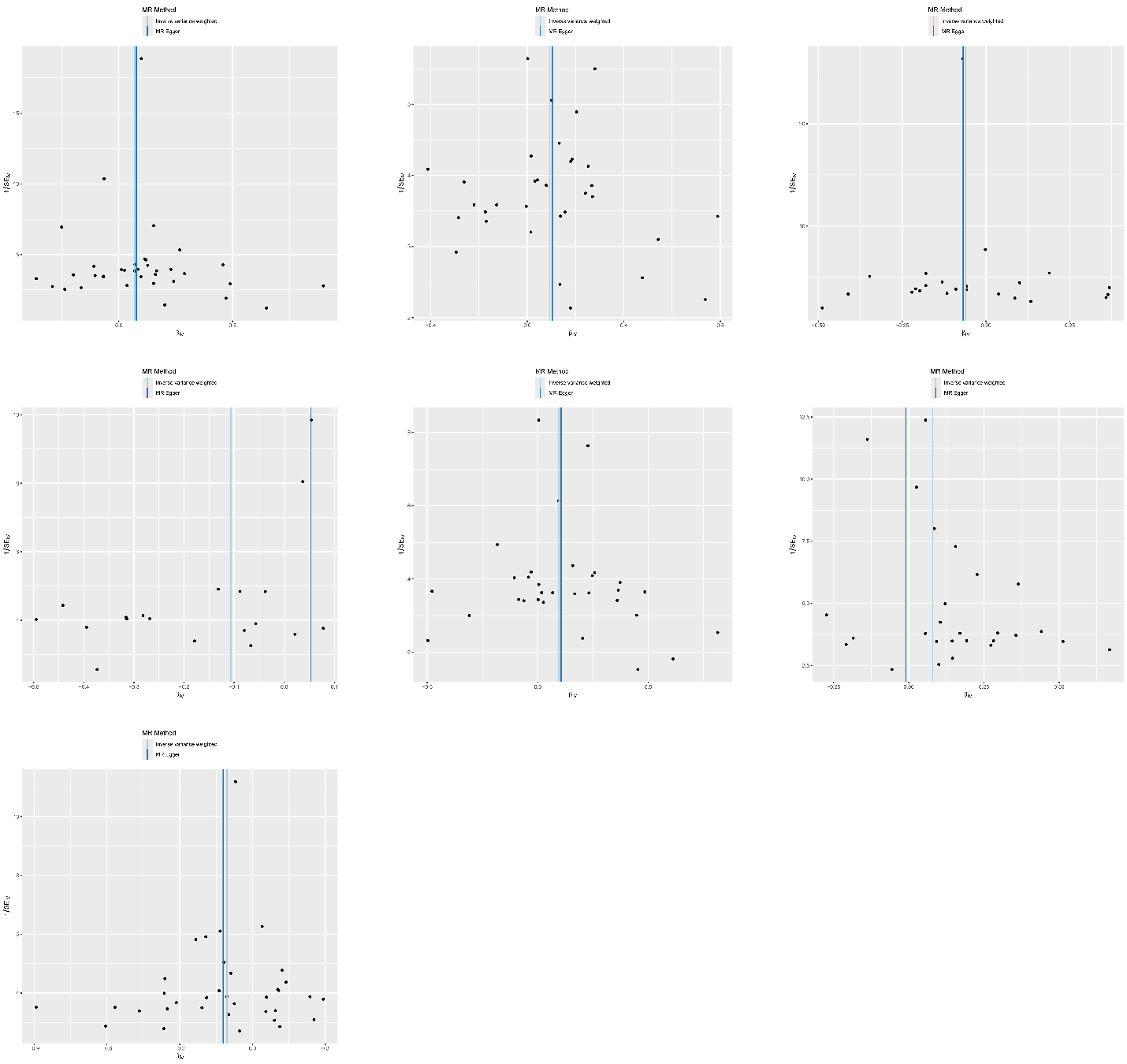


**Supplementary Figure S1c:** The leave‐one‐out analysis of causal impacts of inflammatory cytokines on tinnitus.


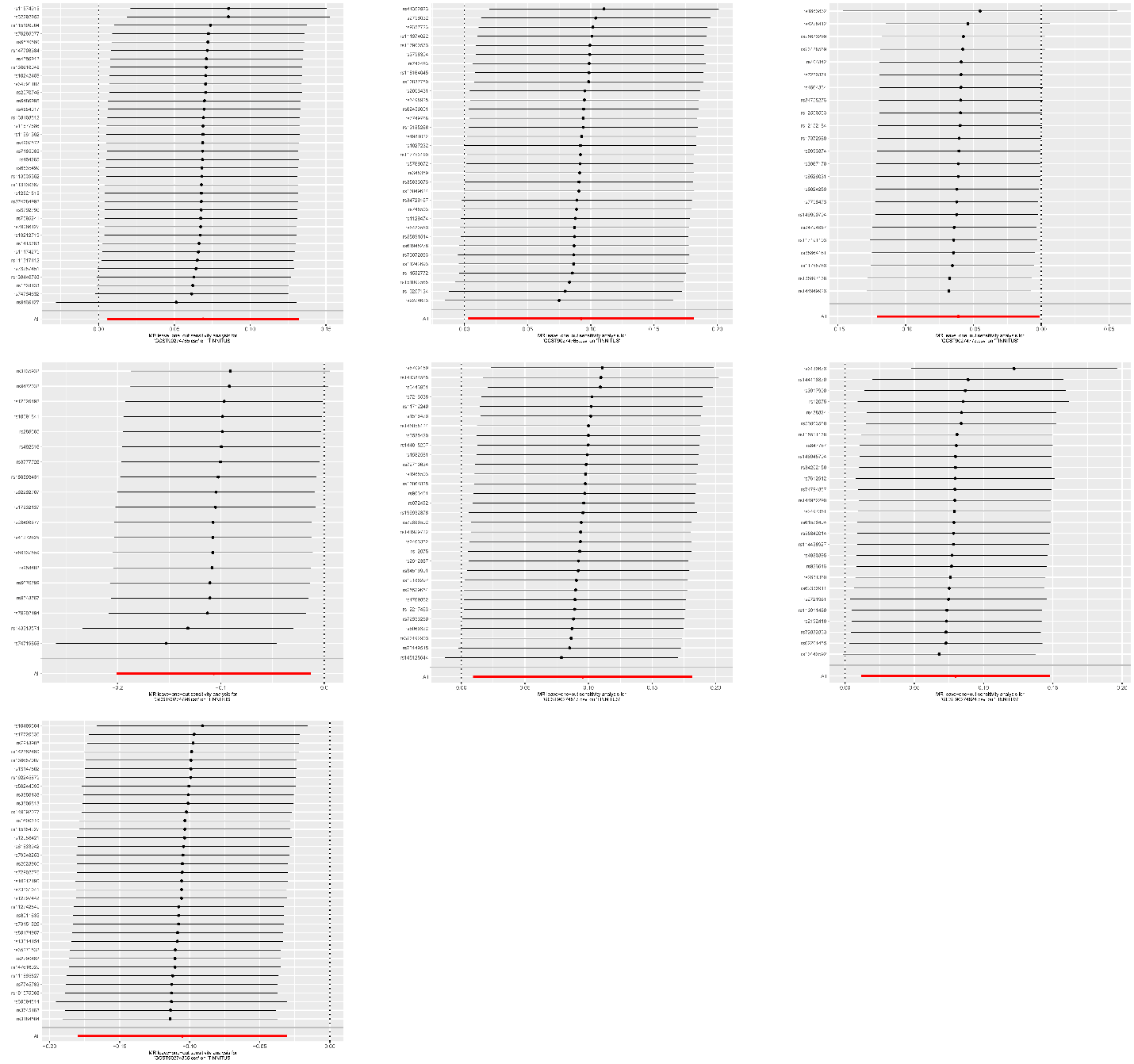


**Supplementary Figure 2**

**Supplementary Figure S2a:** Scatter plots of the causal association tinnitus and inflammatory cytokines using MR study. The two-sample MR analyses were conducted using the MR-Egger, Inverse variance weighted, weighted median, simple mode and weighted mode. The estimated MR effect per method is depicted by the slope of each line.


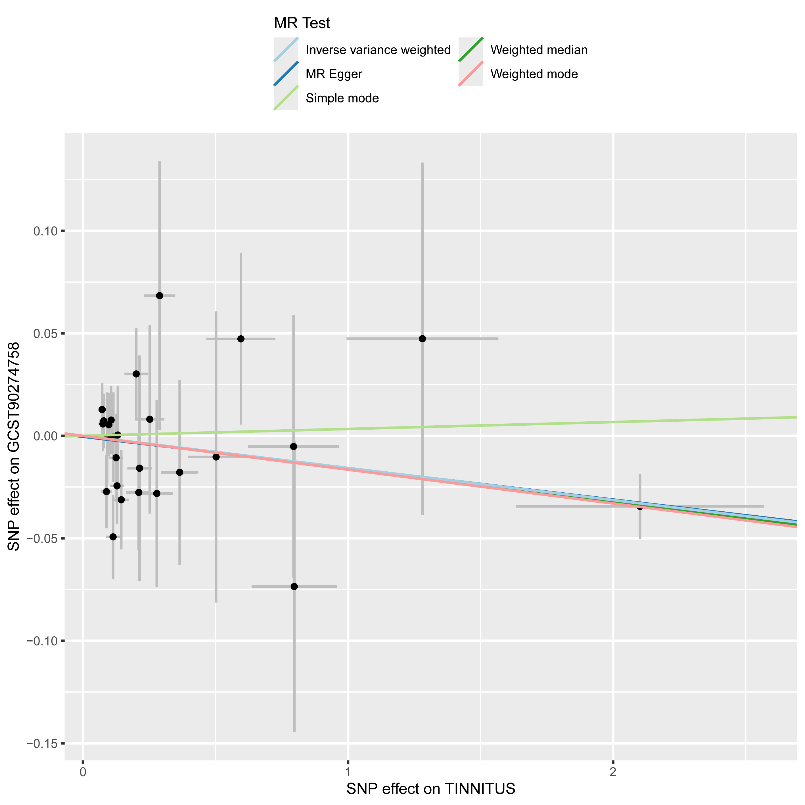


**Supplementary Figure S2b:** Funnel plots of the causal association between tinnitus and inflammatory cytokines using MR study.


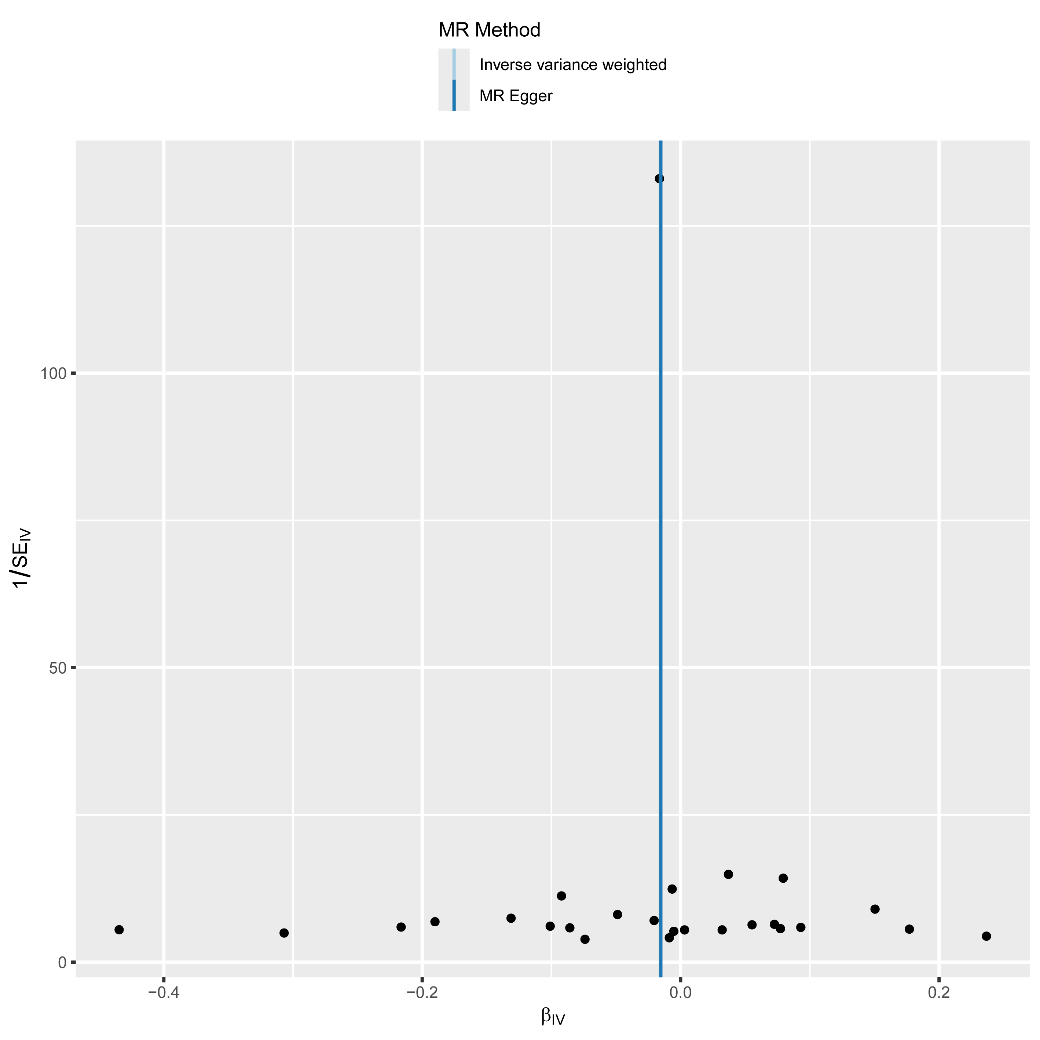


**Supplementary Figure S2c:** The leave‐one‐out analysis of causal impacts of tinnitus on inflammatory cytokines.


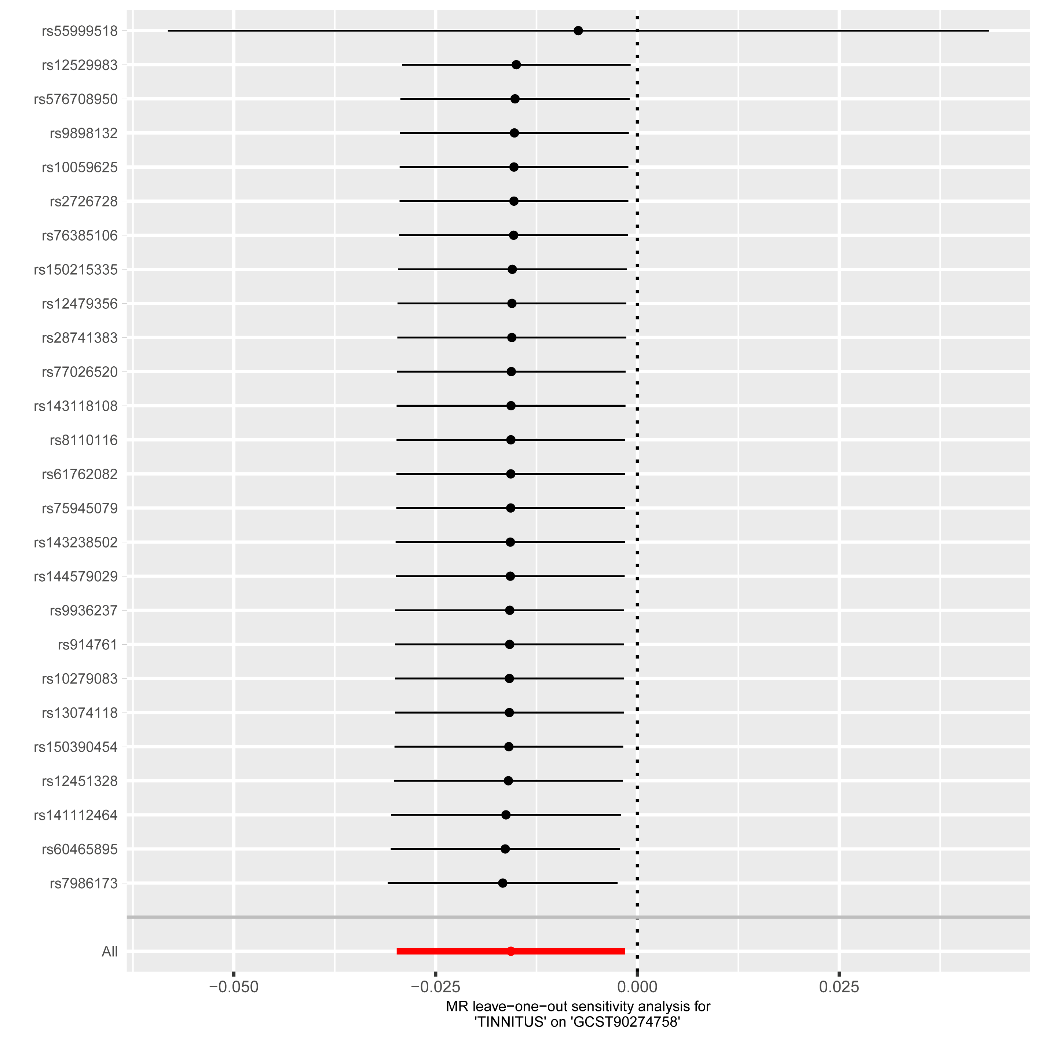

Supplement: Supplementary file 1 — Supporting Fig.1a: Scatter plots of the causal association between inflammatory cytokines and tinnitus using an MR study. Supporting Fig.1b: Funnel plots of the causal association between inflammatory cytokines and tinnitus using an MR study. Supporting Fig.1c: The leave‐one‐out analysis of causal impacts of inflammatory cytokines on tinnitus. Supporting Fig.2a: Scatter plots of the causal association between tinnitus and inflammatory cytokines using an MR study. Supporting Fig.2b: Funnel plots of the causal association between tinnitus and inflammatory cytokines using an MR study. Supporting Fig.2c: The leave‐one‐out analysis of causal impacts of tinnitus on inflammatory cytokines. [file BRB3-15-e70699-s001.docx]
